# An Undefined Interaction between Polyamines and Heat Shock Proteins Leads to Cellular Protection in *Plasmodium falciparum* and Proliferating Cells in Various Organisms

**DOI:** 10.3390/molecules28041686

**Published:** 2023-02-10

**Authors:** Xolani H. Makhoba, Rino Ragno, Annette Kaiser, Enzo Agostinelli

**Affiliations:** 1 Department of Biochemistry, Microbiology, and Biotechnology, School of Molecular and Life Science, University of Limpopo, Turfloop Campus, Sovenga 0727, South Africa; 2 Rome Center for Molecular Design, Department of Drug Chemistry and Technology, Sapienza University, P. le A. Moro 5, 00185 Rome, Italy; 3 Medical Research Centre, University of Duisburg-Essen, Hufeland Strasse 55, 45147 Essen, Germany; 4 Department of Sensory Organs, Sapienza University of Rome, Policlinico Umberto I, Viale del Policlinico 155, 00161 Rome, Italy; 5 International Polyamines Foundation ‘ETS-ONLUS’, Via del Forte Tiburtino 98, 00159 Rome, Italy

**Keywords:** polyamines, heat shock proteins, *Plasmodium falciparum*, plants and drug discovery, cancer

## Abstract

Environmental stimuli can distress the internal reaction of cells and their normal function. To react promptly to sudden environmental changes, a cascade of heat shock proteins (Hsps) functions to protect and act as housekeepers inside the cells. In parallel to the heat shock response, the metabolic polyamine (PA) status changes. Here, we discuss possible ways of putative interactions between Hsps and polyamines in a wide lineage of eukaryotic model organisms with a particular focus on parasitic protozoa such as *Plasmodium falciparum* (*P. falciparum*). The supposed interaction between polyamines and Hsps may protect the parasite from the sudden change in temperature during transmission from the female *Anopheles* mosquito to a human host. Recent experiments performed with the spermidine mimetic inhibitor 15-deoxyspergualine in *Plasmodium* in vitro cultures show that the drug binds to the C-terminal EEVD motif of Hsp70. This leads to inhibition of protein biosynthesis caused by prevention of eIF5A2 phosphorylation and eukaryotic initiation factor 5A (eIF5A) modification. These observations provide further evidence that PAs are involved in the regulation of protein biosynthesis of Hsps to achieve a protective effect for the parasite during transmission.

## 1. Introduction

**Figure 1:**
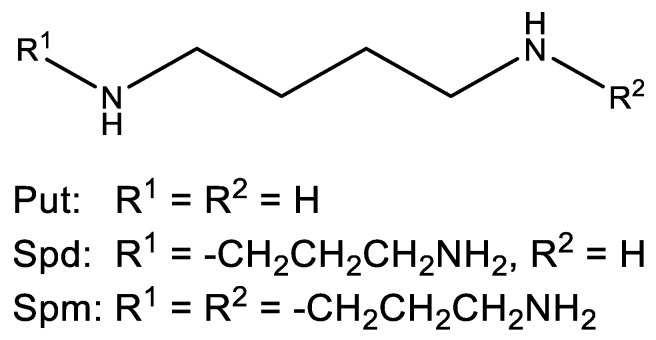
Chemical structures of the main polyamines.

Polyamines (PAs) are ubiquitous positively charged amines bearing more than two amino groups which can be found in all organisms. They play a crucial role in many biological functions including gene regulation, cell growth and differentiation. Mostly important are putrescine (Put), spermidine (Spd) and spermine (Spm) (Figure 1). Generally, PAs are highly reactive polycations, and their biogenesis has been extensively studied [1]. The homeostasis of PAs in the cells depends on their biosynthesis, turnover, export, and transport, including their structural modification through mechanisms such as acetylation and other biochemical conjugations. PAs are ubiquitous molecules that show various covalent and non-covalent interactions in eukaryotic cells. At physiological pH, PAs are positively charged, which makes them interact with many negatively charged macromolecules such as DNA, RNA, phospholipids, chromatin, and proteins, revealing their biogenic potential and their structural properties [2]. Therefore, PAs exert relevant functions in the processes of mRNA transcription and splicing, as well as stabilization of molecular secondary structures, for instance, those of DNA and RNAs. PAs seem to influence the biosynthesis or regulation of certain proteins that are involved in stress-related conditions known as heat shock proteins (Hsps) [3]. The way of this crosstalk is still unknown. It has been shown that polyamine depletion is associated with a decrease of particular Hsps.

Hsps are expressed under stress conditions, e.g., cold [4], UV-light [5], wounding [6] and tissue remodeling [7]. One of their main biological functions is to act as chaperones stabilizing protein structure of de novo synthesized proteins. Moreover, Hsps can also refold proteins damaged by environmental stress [8]. In summary, they function as guardians of proteostasis. Expression of Hsps is transcriptionally regulated and controlled by a heat shock response factor (HSF) [8]. The nomenclature of Hsps is associated with their molecular weight. Hsps with a molecular weight of 60, 70 and 90 kDa belong to families that have been intensively studied [9].

The first evidence for crosstalk between PAs and Hsps was observed after depletion of PAs in a cell line derived from rat hepatoma, which resulted in inhibition of Hsp70 expression [2]. Similar observations were obtained in plants such as *Arabidopsis thaliana* [10], when supplementation with Spm improved the expression of Hsp90 and, in consequence, the enhancement of heat tolerance. In contrast, Hsp90 inhibition increased the polyamine levels of Spd and Spm conjugates. In conclusion, these first experiments suggested an undefined crosstalk between PAs and Hsps leading to a shift in the metabolic state of PAs [10]. Some other studies in plants have shown that PAs exhibit chaperone characteristics and can regulate the synthesis of Hsps [11,12,13]. Thus, the interplay between polyamine modulated Hsps with chaperone activity might have an enormous impact on food production in the future to overcome the needs for an enormously growing world population, in particular in sub-Saharan Africa where climate change has reduced crop production. In this context, experimental observation shows that increasing polyamine contents enhances stress tolerance [14,15,16,17]. Therefore, we decided to study their purported cooperation in a broad lineage of eukaryotic organisms such as quickly proliferating cancer cells, plants and *P. falciparum*, representing the phylum *Apicomplexa*, which evolutionary derives from plants due to the occurrence of an apicoplast [18] with chloroplast origin. Asynchronous nuclear cycles in multinucleated *P. falciparum* facilitate rapid proliferation, as in cancer cells. A common principle of interaction between PAs and Hsps over a broad lineage of organisms would have an enormous translational impact. Since parasites and cancer cells use rapid proliferation to protect themselves against environmental stress, a delineation of the molecular level of this crosstalk would cause significant progress in drug development to improve the chemotherapeutic therapy of either malaria or cancer.

Although 50 years after the establishment of the WHO’s Global Malaria program has passed, malaria is still a worldwide problem of clinical significance causing an estimated 627,000 deaths in 2020 according to a WHO report published in 2021 [19]. Between 2000–2019 death numbers decreased, but the COVID-19 pandemic led to an increase of 12% of deaths in 2020. The death rate of children under the age of 5 slightly decreased, but remained at 72% in 2020 [19]. Malaria is a poverty-related disease, and often accompanied by malnutrition. Eradication of malaria is a major problem, since its causative agent, *P. falciparum*, has a complex life cycle and can remain latent within the human host [19]. The intraerythrocytic life cycle of *P. falciparum* is dynamic, with the parasite undergoing numerous morphological and physiological transformations throughout infection. Whilst actively dividing, the parasite can generate 36 daughter parasites within 2 days. Innumerable parasitic cellular processes offer diverse chemotherapeutic targets for the inhibition of parasite replication and, thereby, abrogation of the disease [20]. A variety of metabolic pathways have shown to be resources for targets in malaria chemotherapy, e.g., vitamin B biosynthesis [21], isoprenoid biosynthesis [22], glucose metabolism, i.e., glucose-phoshate-dehydrogenase [23], and the biosynthesis of PAs [24]. However, the problem of resistance still remains. To interrupt the interaction between Hsps and PAs in *Plasmodium* [25] with small molecules will be an important issue for drug discovery in the future.

In recent years, cancer has caused the death of millions of people worldwide. Numerous studies have shown that PAs are involved in the differentiation, proliferation, and growth of cancer cells [26]. Their concentration is much higher in cancer cells than in normal cells [27]. Moreover, Hsps serve as protective molecules, thereby contributing to keeping cancerous cells alive. Here, we review a variety of functions and a possible correlation between PAs and Hsps in parasitic protozoa, plants, and cancer cells to delineate common mechanisms over a broad eukaryotic lineage.

## 2. The Heat Shock Response

Cells respond to environmental stress with an ancient and conserved mechanism, i.e., the heat shock response, to prevent possible damage to proteins. These environmental stress conditions comprise, e.g., immediate temperature increase, toxic substances, UV light, wounding and ischemia [28]. In the first step, heat shock activation factor 1 (HSF-1), a transcription factor, upregulates class 1 of Hsps, the molecular chaperones. This is achieved by binding to heat shock elements in the genes that function as molecular chaperones. These molecular chaperons are involved in refolding of denatured proteins, or in escorting protein misaggregates to degradation pathways, thus preventing an accumulation of misfolded proteins [29]. PAs such as Put, Spd, and Spm are ubiquitous polycations that have numerous covalent and non-covalent interactions in eukaryotic cells. In this context, it is that PAs influence the DNA binding of heat shock factors and transcription factors [30]. Spm depletion caused by α-difluoromethylornithine (DFMO) decreased the activation of Hsp90 after heat-shock in a Fao cell line and could be restored by replenishment of spermidine [31]. These results can be explained by the physico-chemical properties of Pas, which favor an interaction with anionic macromolecules such as DNA, RNA [32,33]. PAs exert main functions such as mRNA transcription and splicing, as well as stabilisation of molecular secondary structures, for instance of DNA and RNA [33]. These physico-chemical properties might explain a modulated regulation of Hsps by PAs.

## 3. A Common, Regulatory Mechanism between Polyamines and Heat Shock Proteins Might Be Exploited in Crop Production

Unsustainable growth of the world population will endanger water and food supply during coming years. In this context, different environmental conditions on plants and crops will have an impact on food and nutrition Therefore, basic research has to be fostered to study and understand the molecular mechanisms of interactions between Hsps and PAs in different plants.

**Figure 2:**
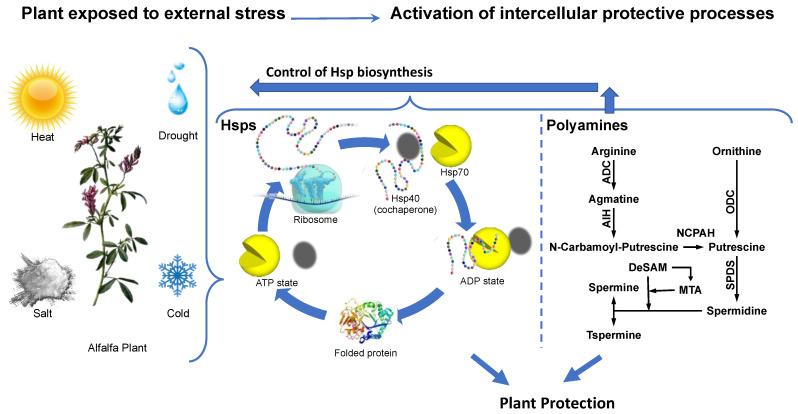
Benefits of heat shock proteins and polyamines in crop production. External conditions such as heat, drought, salt and cold, activate the expression of Hsps and synthesis of PAs as protective factors under stressful conditions. The folding process of proteins is driven by the ATP/ADP cycle of heat shock protein 70 (Hsp70) in cooperation with the Hsp40 co-chaperone. Biosynthesis of PAs promotes cell differentiation, proliferation, and cell growth. An undefined, molecular mechanism, triggered by PAs, leads to a control of Hsp biosynthesis and results in plant protection. Abbreviations: ADC, S-Adenosylmethionine decarboxylase, ODC, ornithine decarboxylase, AIH, agmatine iminohydrolase, NCPAH, N-carbamoylputrescine amidase, DeSAM, decarboxylated SAM, MTA, 5′-methyl-thioadenosine, SPDS, Spermidine synthase.

Environmental changes pose a challenge to plant growth and could lead to detrimental consequences, such as food shortage. Biotic and abiotic conditions can affect the growth of plants, including water shortage and extreme heat conditions (Figure 2 left panel). In recent years global warming has caused great damage to the agricultural sector [34] in many African countries, decreasing their productivity. This has led to the outbreaks of various diseases, as well as malnutrition. PAs have been shown to play an essential role in stress responses of plants. For example, in alfalfa (Figure 2 left panel), it has been reported that PAs, such as Put, Spd, and Spm (Figure 2, right panel), influence the synthesis of Hsps (Figure 2, medium panel) to protect the plants under stress conditions [35]. This crosstalk between Hsps and polyamines is also observed after transformation of polyamine biosynthetic genes encoding arginine decarboxylase (ADC), ornithine decarboxylase (ODC), *S*-adenosylmethionine decarboxylase (SAMDC) or Spermidine synthase (SPDS) [36] to achieve protection after abiotic stress signals.

A more detailed, molecular analysis for the interaction between PAs and Hsps has been performed in *Arabidopsis thaliana* [37]. Exogenous Spm increased the transcription of Hsp90 and subsequently enhanced heat tolerance. Interestingly, silencing of the four cytosolic Hsp90 genes resulted in a significant shift of the metabolic status of polyamines. Several-fold higher levels of soluble spermidine (S-Spd), acetylated Spd (N^8^-acetyl-Spd) and acetylated spermine (N^1^-acetyl-Spm) were detected in the silenced transgenic *Arabidopsis thaliana* lines [37]. A loss-of-function mutant in one of the Hsps encoding genes led to an increase of polyamine oxidase activity (PAO) with a significant release of hydrogen peroxide (H_2_O_2_). Moreover, genetically manipulated low expression levels of PAO resulted in thermotolerant tobacco plants [38]. Such findings suggest cross-talk between Hsp90 and PAO to orchestrate PA acetylation, oxidation, and PA/H_2_O_2_ homeostasis which results in protection and a benefit for crop production [38].

## 4. A Heat Shock Affects the Regulation of Polyamines on the Transcriptional and Translational Level in Plants

**Figure 3:**
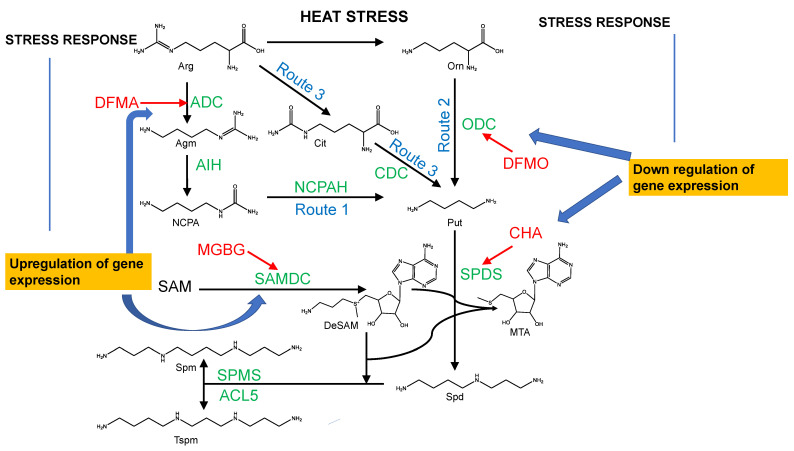
Abiotic Stress in plants affects the polyamine pathway at the transcriptional and translational level. PAs such as putrescine (Put), spermidine (Spd), spermine (Spm) and thermospermine (Tspm) are formed either from arginine (arg) or ornithine (orn) by three different routes [39]. The pathway, initiated by arginine decarboxylase (ADC), leads to the formation of agmatine (Agm), which is converted by agmatine iminohydrolase (AIH) to N-carbamoylputrescine (NCP). NCP is hydrolized by N-carbamoylputrescine hydrolase (NCPH) to Put (Route1). Arg can be converted to Orn by arginase (Route 2) or to Citrulline (Cit) (Route 3), which is decarboxylated further by citrulline decarboxylase (CDC) to putrescine. Important inhibitors of the pathway are DL-alpha-difluoromethylarginine (DFMA), alpha-difluoromethylornithine (DFMO), methylglyoxalbisguanylhydrazone (MGBG), and cyclohexylamine (CHA). After heat shock, or under abiotic stress, plants react with stress responses that can lead to up- or down regulation of gene expression (left and right panel of Figure 3). In *Lycopersicon esculentum,* ADC, SAMDC and ODC1 are the most upregulated genes, while SPDS is downregulated.

Having analyzed the benefits of an undefined interaction between PAs and Hsps in crop plants, we collected information about the regulatory mechanisms under heat stress in plants beyond. Putrescine is the main, central precursor of the polyamine pathway in plants and is formed either from arginine or ornithine. There are three different routes to form Put in plants [10] (Figure 3). In the first route arginine (Arg) is decarboxylated by arginine decarboxylase (ADC) to form agmatine (Route1) which is converted to N-carbamoyl putrescine (NCP) by agmatine iminohydrolase (AIH). Next, NCP is hydrolysed to putrescine by N-carbamoylputrescine amidohydrolase (NCPH). This is the main route to synthesize Put in plants. Within route 2, ornithine is produced from arginine by arginase and then decarboxylated by ornithine decarboxylase (ODC) to Put. In the third route, Arg is first converted to citrulline (Cit), which is then decarboxylated by citrulline decarboxylase (CDC) to form Put. (Figure 3) Spermidine synthase (SPDS), an important aminopropyltransferase, catalyzes the reaction from Put to Spd adding an aminopropyl moiety from decarboxylated S-adenosylmethionine (dcSAM) to Put. The most common PAs in higher plants are Put, Spd, Spm, thermospermine (Tspm) and cadaverine (Cad) [39]. In recent years, different specific inhibitors against the rate limiting enzymes of polyamine synthesis have been identified such as DL-α-difluoromethylarginine (DFMA), an irreversible inhibitor of ADC or DL-α-Difluoromethylornithine (DFMO) which irreversibly targets ODC (Figure 3). Methylglyoxalbisguanylhydrazone (MGBG) was the first competitive inhibitor of S-Adenosylmethionedecarboxylase (SAMDC), a branchpoint enzyme in the polyamine pathway. The drug is more toxic than DFMO since it reduces the availability of decaboxalated methionine for protein synthesis. Cyclohexylamine (CHA) competitively inhibits SPDS. It occupies the polyamine binding site of SPDS where it binds at the bottom of the active site with its amine group which is placed in analogy to its natural substrate.

In plants, the response to abiotic stress affects gene expression of the polyamine pathway at the transcriptional and translational level. The impairment of polyamine biosynthetic genes and their anabolic enzymes have recently been shown under heat stress in *Lycopersicon esculentum* (tomato) leaves [40]. Remarkably, higher transcript levels were monitored for *SAMDC1*, *SAMDC2* (S-adenosylmethionine decarboxylase) and *ADC2* (arginine decarboxylase) genes in nearly all tissues after HEAT shock while transcript levels of *ADC1,2*, *ODC1,2*, and *SPMS* declined [Figure 3]. From the anabolic enzymes only copper amine oxidase (*CuAO4)* increased during heat stress [41]. In summary, cellular levels of both free and conjugated forms of putrescine and spermidine were found to decrease during heat stress.

Interestingly, plants show a higher number of heat shock factors (HSFs) than humans. For instance, there are 38 HSFs in soybean, 25 in rice, and 21 in Arabidopsis compared to seven in humans [41]. These results suggest the importance of HSFs in plants to react to a variety of stress signals by expression of different HSFs. In plants, the main polyamines, putrescine, spermidine, spermine and thermospermine, regulate the binding of HSFs to heat shock elements, and, in turn, to protection.

A direct link between the polyamine pathway and HSF-1 has recently been shown in IEC-18 rat intestinal epithelial cells. Induction of ODC by glutamine increases expression of Hsp70 and Hsp25 [42]. Moreover, the inhibitor DFMO significantly decreased binding of HSF-1 to a synthetic oligonucleotide containing a heat shock response element. These results suggest that glutamine.-dependent induction of ODC may facilitate the binding od HSF-1 to the responsive heat shock elements (HE) [42]. This interaction has not been investigated in plants, but might be transferable.

**Figure 4:**
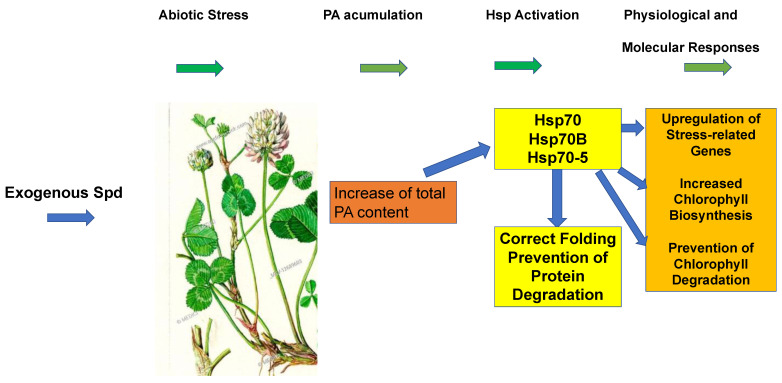
Exogenous spermidine supplementation in white clover supports stress tolerance after abiotic stress. Spd-treated white clover shows increased PA accumulation and Hsp activation under abiotic stress conditions with subsequent physiological and molecular responses of the plant, which finally lead to protection.

There are many examples that prove the participation of PAs in stress tolerance under abiotic conditions. In a recent review published by Alcázar [43], the protective effect of exogenous PAs and genetically altered PA biosynthesis under abiotic stress were discussed. Exogenous application of polyamines alters gene expression. Among them are genes controlling the synthesis of protective metabolites such as PAs. In this context, PAs can be considered as metabolic hallmarks. PAs are beneficial for protein homeostasis, detoxification of reactive oxygen species (ROS) and molecular chaperone activity under stress conditions. Recently, the spermidine synthase2 gene from tomato was overexpressed in tomato seedlings to study the protective function of triamine spermidine under saline-alkali stress [44]. It was shown that the overexpressed spermidine synthase significantly reduced the Na^+^/K^+^ ratio, relative electrical conductivity, O_2_^·−^, H_2_O_2_, and malondialdehyde content. The improved resistance against salt-alkaline stress of tomato seedlings was associated with a significant increase in endogenous, free PA content, thereby improving resistance of tomato seedlings against salt alkaline stress. Lu et al. (2020) published a more in-depth study of exogenous supplementation with spermidine in white clover [45] (Figure 4). The results showed that exogenous spermidine alleviated levels of total, endogenous PAs. In turn, this led to increased expression levels of different Hsps70, causing a variety of physiological and molecular responses. Notably, chlorophyll biosynthesis was upregulated in Spd-treated white clover, supporting an interaction between PAs and Hsp networks of an undefined mechanism.

## 5. Heat Shock Proteins in Malaria Parasites: Biological Function and Pharmacological Relevance

Malaria parasites undergo fast and extensive proliferation, as in cancer cells. For this reason, we selected *P. falciparum* as a model organism to study an interaction between PAs and Hsps. *P. falciparum* is exposed to a wide range of temperature fluctuation during its life cycle. During transmission from the arthropod vector to the human host, it encounters around a 12 °C switch in the environmental temperature. Moreover, the parasite must adapt to temperature fluctuations due to the febrile episodes that occur during the clinical manifestation of the disease. Considering the repeated heat stress conditions by the parasite during its life cycle, the presence of a robust heat shock response machinery is essential for its survival. Apart from the protection of the parasite, Hsps play an important role in the pathological development of malaria, which is mainly due to their export in the cytosol of the infected erythrocyte [25]. Moreover, some of the Hsps determine the virulence of the parasite. The *Plasmodium* genome consists of 59 Hsps. Forty-nine of them belong to the Hsp40 family [25] representing the largest family. Members of this family mainly function as molecular chaperones and are involved in malaria pathogenesis, in particular in immune modulation of the host cell. Molecular chaperones play an important role during de novo protein synthesis, and facilitate the subsequent trafficking of proteins to desired destinations. They are further responsible for the correct folding of proteins into their native three-dimensional conformations. Moreover, they support the assembly of multi-protein complexes [46]. All six *Pf*Hsps90 are essential for parasite survival and virulence. They play a main role in refolding of misfolded proteins that are detected by Hsp40. This has been shown by a knockout mutant of cytosolic *Pf*Hsp90 [46]. Members of the Hsp90 family interact with functional partners forming a network. Moreover, small, pharmacologically active inhibitors such as epigallocatechin-3-gallate have been identified [47] that interrupt the association with other Hsps either from the parasite or the human host. There are only four *Pf*Hsps70 in the *Plasmodium* genome. Apart from their function as chaperones, they are considered as nucleotide exchange factors [48]. Interestingly, a knockout of exported PfHsp70-z compromised *P. falciparum* virulence [44]. This detrimental effect is attributed to prevention of its association with PfHsp70-1 to stimulate its ATPase activity. In summary, the results suggest a network of an interactome of *Pf*Hsps70 in *Plasmodium*. To interrupt the interactome of the exported plasmodial Hsps will be important for the future in drug discovery. One key to identifying selective inhibitors could be the targeting of the regulatory motifs, such as the C-terminal -EEVD motif within the PfHsps90 required for the formation of multi-chaperone complexes.

Surprisingly, only a homologue of a heat shock factor binding protein (*Pf*HSPB) has been identified in *Plasmodium* [49], while to date an HSF has not been discovered. *Pf*HSPB plays a role as a negative regulator in the heat shock response since it appears in the recovery phase. The protein is expressed in trophozoites, and its secondary structure is highly conserved with a high helical content, forming oligo monomers. The protein is localized in the cytosol of the parasite. Upon heat shock it is translocated to the nucleus. *Pf*HSPB might be the key to identify the heat shock response factor in *Plasmodium.*

**Figure 5:**
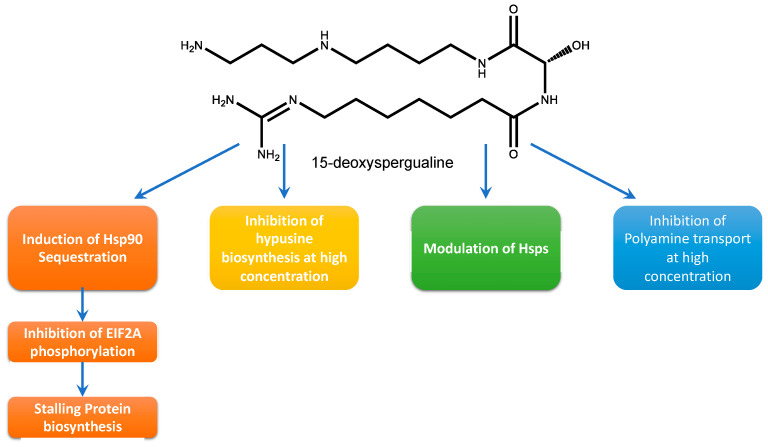
15-deoxyspergualine and its mode of molecular action.

A link between the polyamine pathway and Hsps has been indirectly demonstrated by the drug 15-deoxyspergualine (DSG) [50] (Figure 5). The drug is an immunosuppressive administered in tumor therapy, and has antimalarial properties. DSG has an impact on a variety of nuclear pathways, i.e., protein biosynthesis, polyamine biosynthesis and polyamine transport [51]. Hsps are considered to play a role in protein translation. One effect of DSG is the stalling of protein biosynthesis, which can be attributed to Hsp90 sequestration and silencing of a protein kinase necessary for phosphorylation of eukaryotic initiation factor eIF2A, which promotes protein biosynthesis [50]. Moreover, the drug modulates the expression pf Hsp70-1 by binding to an EEVD motif at the C-Terminus of the protein [52]. Apart from eIF2A, DSG competitively inhibits deoxyhypusine synthase (DHS), the regulatory enzyme of the hypusine pathway, and thereby affects protein translation through eIF5A inhibition [53].

**Figure 6:**
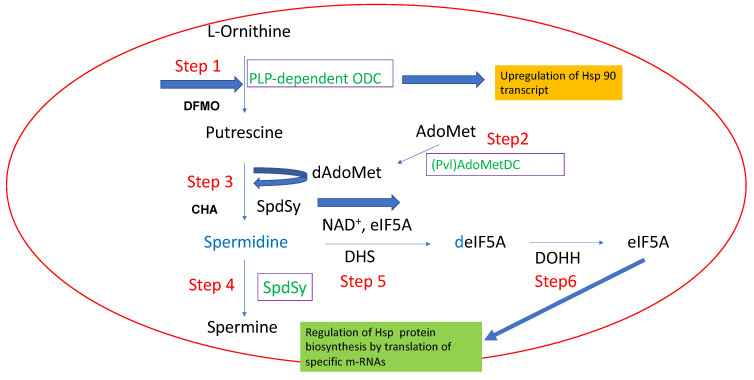
The polyamine pathway in Plasmodium species and its link to Hsps. Ornithine decarboxylase is involved in the upregulation of Hsp90 transcripts, while hypusinated eIF5A regulates the biosynthesis of Hsps.

PAs in *Plasmodium* are exclusively synthesized from the amino acid ornithine (orn) [54] (Figure 6) in contrast to mammalian cells that either use orn or arg as precursor molecules of the pathway. The biosynthetic route starts with the decarboxylation of orn catalyzed by pyridoxal 5′-phosphate (PLP)-dependent ornithine decarboxylase (ODC), resulting in the formation of putrescine (Put) (step1).

Transcriptional profiling was performed in the malaria parasite after polyamine depletion with DFMO [55], irreversibly inhibiting ODC. An analysis of the most upregulated transcripts detected Hsp70 with a 3–5 fold increase in transcript level, confirming a direct link between the polyamine pathway and Hsp regulation. Spermidine (Spd) synthase (step 3) transfers the aminopropyl moiety delivered by pyruvoyl (*Pvl*)-dependent SAM decarboxylase (AdoMetDC) (step 2) to Put, resulting in the formation of spd. Although a genomic locus for Spd synthase has not been identified in *Plasmodium*, spermine (Spm) can be formed by Spd synthase (step 4). A recent study investigated the expression of transcripts under polyamine perturbation with cyclohexylamine (CHA), an inhibitor of spermidine synthase in *Plasmodium* [56]. The authors detected significant changes in transcripts of genes involved in purine metabolism, arginine and proline metabolism but not in *Hsp*-encoding transcripts.

Spd is the substrate of deoxyhypusine synthase (DHPS) (step 5), which transfers the aminobutyl moiety in a NAD^+^-dependent reaction to a specific lysine residue in eukaryotic initiation factor 5A (eIF5A). In a subsequent step (step 6), the side chain of deoxyhypusinated eukaryotic initiation factor 5A is hydroxylated by the enzyme deoxhypusine hydroxylase (DOOH). Moreover, DOHH from *Plasmodium* has peculiar features, i.e., it has a dioxygen-bound di-iron core during O_2_-activation that is unrelated to 2-oxoacid-dependent dioxygenases [57] and EZ-HEA-like repeats [58], suggesting an interaction with other proteins that might be Hsps. An effect of DSG on the polyamine pathway, i.e., the biosynthesis of hypusine in *Plasmodium* (Figure 6), was only observed at high concentrations of the drug ranging from 250 μM to 1 mM [50], suggesting a link to protein biosynthesis via eIF5A. It is tempting to speculate that eIF5A might be involved in translation of specific Hsps encoding mRNAs. However, the effect of DSG on protein translation through eIF5A is significantly weaker compared to that of eIFA2 through Hsp90 sequestration.

## 6. Heat Shock Proteins in Cancer Cells

Cancer cells do not respond to signals that normally control growth and death. DNA changes in tumor suppressor genes are responsible for this rapid and uncontrolled growth. Since rapid proliferation of cancer cells resembles the rapid growth of the malaria parasite, it is of enormous interest to understand the interaction between Hsps and PAs in controlling the biological development of cancer cells.

Apart from their biological function in cancer cell development, Hsps are involved in multiple clinical usages as biomarkers and are potential, therapeutic targets in cancer chemotherapy [59]. These Hsps include Hsp27, Hsp40, Hsp60 and Hsp90. Their biological function is attributed to their activity as molecular chaperones controlling protein homeostasis by promoting the correct folding and refolding of denatured proteins. Some of the Hsps fulfill peculiar functions in cancer cells, such as Hsp60, which is localized in the mitochondria and functions as a promotor and suppressor in growing cancer cells [59]. In contrast, Hsp27 represents a small (12–43 kDa), ATP-independent chaperone which is responsible for tumor initiation, programming of cancer stem cells and metastasis [60] affecting key determinants for overall survival. However, its over-expression in cancer cells leads to poor prognosis for patients. Hsp40s are classified into DnaJA, DnaJB and DnaJC, and are mainly involved in cancer progression and metastasis [60]. Hsp70s and Hsp90s are of high impact for protein folding and determination of malignancy and progression. All HEAT-shock responses are controlled by the HSF-1 protein as a master regulator [60]. In conclusion, Hsps exert a cytoprotective function in various types of cancer cells during their biological development.

Apart from their function as chaperons, Hsps can alter numerous signaling pathways. While Hsp27 interferes with the Hippo pathway, Hsp40 changes the ERK pathway and Hsp60 affects the TOR pathway. Notably, Hsp70 interacts with a variety of intracellular pathways such as JAK/STAT, HIF1α and extracellular pathways.

Hsps can also confer resistance in cancer chemotherapy. Here, we discuss different mechanisms that are used by Hsps to inactivate important drugs in anticancer chemotherapy. Hsp27 is a pivotal candidate since it is synergistically involved in intracellular and extracellular pathways, i.e., NF- κB transactivation and induction of different, mitochondrial, apoptotic pathways. This has been shown in squamous cell cancer of the tongue (SCCT) [61] where chemoresistance is a common phenomenon.

A second example of conferring resistance in cancer chemotherapy is the loss of a Hsp40 protein in ovarian cancer cell lines, causing multi-drug resistance against paclitaxel, topotecan and cisplatin [62]. Thirdly, chemoresistance against EGFR tyrosine kinase inhibitors (EGFR-TKIs) such as Erlotinib was observed in patients with non-small-cell-lung cancer (NSCLC) harboring an EGFR T790 M mutation which led to reduced phosphorylation of Hsp90 and final ubiquitination and degradation of the protein [63]. Collectively, chemoresistance in cancer chemotherapy is caused either by loss of gene function of a particular Hsp or the occurrence of mutations in associated, key oncogenic drivers [64] and pathways.

## 7. The Interplay between PAs and Hsps in Cancer Cells can Cause Different Responses Depending on the Microenvironment: From Protection to Aggressiveness

In various cancer types, PA levels are elevated, and dysregulation of PA metabolism has been observed in many neoplastic cells [65]. A recent review strengthens the role of PAs in establishing a tumor-permissive microenvironment by blocking the natural immune checkpoint blockade [66]. Since Hsps also fulfill cytoprotective functions in a variety of tumor cells, it is of great scientific interest to understand the interplay between PAs and Hsps for novel anticancer chemotherapies.

In 1996 Ignatenko and Gerner [67] identified a correlation between PAs and heat shock in cancer cells. The authors discovered a 3–5 fold increase of Spd/Spm N1-acetyltransferase (N1SSAT) when human colon tumor-derived HCT116 cells traversed the log phase and entered the plateau phase. This effect could be reversed by supplementation with exogenous Spd. A second, important finding was that PAs, in particular spermidine, facilitate the binding of HSF-1 and activator protein 1 transcription factors [29], leading to cytoprotection in cancer cells.

Similar observations with therapeutic potential have been made for hypusinated eIF5A in different cancer types. Activation of eIF5A results in an accumulation of cells that promote tumor cell growth and aggressiveness. Remarkably, this includes the eIF5A Hsp27–nuclear factor kB axis [68]. Like eIF5A, Hsp27 is associated with cell proliferation and is highly expressed in many human cancers where it enhances cell survival via activation of NF-κB. Inhibition of DOHH, the second step of hypusine biosynthesis, by ciclopiroxolamine in cervical cancer cells, ameliorated eIF5A biosynthesis and sequentially Hsp27 [69].

In contrast, eIF5A can also exhibit tumor suppressive activity [70] in lymphomagenesis. Because hypusinated eIF5A controls translation of specific mRNAs, it is possible that a set of regulatory proteins is functional, depending on the tumor type and the microenvironment, leading to the opposite biological effect, which is cytoprotection of healthy cells.

## 8. Hsp Inhibitors of Anticancer Chemotherapy might Be Useful for Pharmacological Intervention to Disrupt the Interplay with PAs

It has been shown that Hsp27, Hsp40, Hsp60 and Hsp90 are highly and differentially expressed in cancer cells [60] and are associated with key oncogenic drivers. Accordingly, they are important immunmodulators, which are of advantage for anti-cancer treatment. Hitherto, no direct Hsp inhibitors have been approved and registered. However, one inhibitor, sorafenib (Nexavar^®^), a multi-kinase inhibitor that targets client proteins of Hsps, has gained approval to treat renal, hepatocellular, and thyroid carcinoma [71]. Meanwhile, promising results have been obtained for the three direct Hsp90 inhibitors geldanamycin, gramitinib and shepherdin, which are under clinical evaluation [72]. All of them bind to the N-terminal ATP-binding pocket of the Hsp90 protein. While geldanamycin affects a variety of different cancer types, gramitinib is applied in the treatment of lymphoma. Shepherdin is effective against glioblastoma and lymphoma by inducing apoptosis. In this context, it would be of further clinical interest to investigate whether depletion of PAs could enhance this therapeutic effect, since dysregulation of polyamine metabolism is observed in cancer cells.

Table 1 summarizes the mode of action how Hsps inhibitors target a specific Hsp protein to treat various types of cancer. Quercetin, a natural flavonoid, suppresses the anti-apoptotic effect of Hsp27 [73] in a leukemia cell line in synergism with a short hairpin (sh) RNA. This leads to inhibition of cell proliferation and induction of apoptosis by decreasing the Bcl2-to-Bax ratio. The combined therapy suppresses infiltration of tumour cells and the expression of angiogenesis-associated proteins, i.e., hypoxia-induced factor 1 (HIF1α) and vascular endothelial factor (VEGF). Since PAs promote development of various types of cancer cells, downregulation of their biosynthetic genes might be an excellent strategy that can be combined with a specific Hsp inhibitor.

Myrtocommulone A (MC), a non-prenylated acylphloroglucinol from myrtle (*Myrtus communis*, Myrtaceae), has been identified to target Hsp60 in a discriminative protein fishing approach [74]. MC modulates Hsp60 function in mitochondria. Administration of MC prevents Hsp60-mediated refolding of denatured malate dehydrogenase in a protein folding assay. Moreover, MC administration leads to mitochondrial dysfunction leading to apoptosis in cancer cells [7]. Recently, it was shown that inhibition of EIF-5A induces apoptosis in cardiomyocytes after malaria infection [77] via tyrosine sulfation of the protein [78]. Collectively, the data show that Hsps and hypusinated eIF5A exhibit antiapoptotic effects.

Cantharidin, a monoterpenoid from maylabis, was identified in cell-based screening of a library with commercial and experimental drugs to inhibit Hsp70 in a colorectal cancer line [75]. The drug inhibits HSF1 binding to the promotor of Hsp90. Downstream effects were observed for other proteins, such as BCL-2 inducing apoptotic cell death [75].

Ganetespib is a second-generation Hsp90 inhibitor that exhibits potent cytotoxicity in vitro and demonstrates antitumor activity with promising safety profiles in vivo for a diverse group of cancers [76]. The compound inhibits Hsp90 in a dose-dependent manner in eight thyroid cancer lines. Hsp90 has a variety of client proteins involved in cell cycle progression. It decreased cyclin-dependent kinase 1 and arrested cell cycle progression in G2/M phase [71] and RAS/RAF/ERK and PI3K/AKT/mTOR signaling pathway.

## 9. Conclusions and Perspectives

This review summarizes current available data concerning Hsps interactions with PAs to achieve a common protective effect. Data collection was performed in a wide lineage of eukaryotic cells with a particular focus on parasitic protozoa such as *Plasmodium*. In crop plants and plants under abiotic stress conditions, the interplay between PAs and Hsps leads to a set of protective, physiological responses due to an accumulation of PAs and an activation of Hsps. In *Plasmodium*, modified eIF5A is partly involved in the interaction with heat-shock proteins to promote cell proliferation and protection during stress conditions in its life cycle. This effect might be explained by translation of a set of specific Hsps mRNAs. In cancer cells, the crosstalk between Hsps and PAs leads to progression of tumors in a variety of cancer types, which is non-protective for the patient. Apart from promoting tumor growth, hypusinated eIF5A can lead to tumor suppression in lymphomagenesis and to subsequent protection. Thus, understanding the molecular interaction of PAs and Hsps is important in urgent experimental approaches to advance this interesting field. Interruption of this interaction might be beneficial for the treatment of various tumors. In this context, a combined chemotherapy of already promising direct Hsp inhibitors and PA antagonists might be a novel strategy.

## Figures and Tables

**Table 1:** Representative heat-shock protein inhibitors for anticancer therapy.

Target	Compound	Cancer	Mode of Action
Hsp27	Quercetin 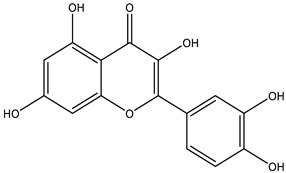	Leukemia	⇒ Induction of Apoptosis ⇒ Decrease of Bcl2-to Bax ratio ⇒ Blockade of the cell cycle at the G1 phase [73]
Hsp60	Myrtucommulone A 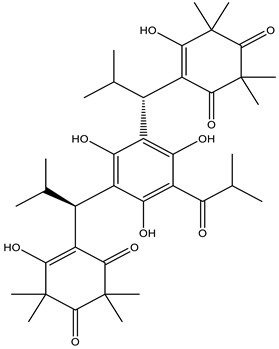	Leukemia	⇒ Release of cytochrome C from mitochondria ⇒ Induction of apoptosis [74]
Hsp70	Cantharidine 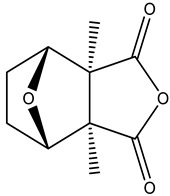	Colorectal Cancer	⇒ Blockage of HSF1 binding to HSP70 promoter ⇒ Induction of apoptosis via the inhibition of heat shock response and HSP70 expression [75]
Hsp90	Ganetespib 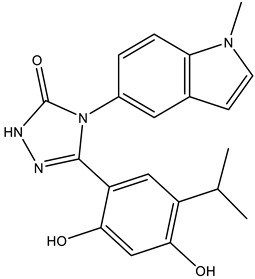	Thyroid, breast, lung and ovarian cancer	⇒ Inhibition of cell proliferation metastasis ⇒ Induction of cell cycle arrest ⇒ Enhancement of apoptosis ⇒ decrease of tumor growth [76]

## Data Availability

Data availability statements were all added in the whole text, in the template.
